# Use of a Head-Mounted Assisted Reality, High-Resolution Telemedicine Camera and Satellite Communication Terminal in an Out-of-Hospital Cardiac Arrest

**DOI:** 10.1016/j.mcpdig.2024.09.002

**Published:** 2024-10-09

**Authors:** Christopher S. Russi, Sarayna S. McGuire, Aaron B. Klassen, Kate M. Skeens, Kate J. Arms, Lindsey D. Kaczmerick, Patrick J. Fullerton, Louis M. Radnothy, Anuradha Luke

**Affiliations:** a Department of Emergency Medicine, Mayo Clinic, Rochester, MN; b Mayo Clinic Ambulance Service, Rochester, MN; c Orlando College of Osteopathic Medicine, Orlando, FL; d Uniformed Services University of the Health Sciences, San Antonio, TX

## Abstract

Mayo Clinic Ambulance Service is testing a novel combination of technologies to enhance the ability to provide prehospital telemedicine connecting physicians with paramedics. Mayo Clinic Ambulance Service partnered with start-up company OPTAC-X to field test a novel head-mounted video camera connected with a satellite communications terminal to bring medical control emergency medicine physicians to the patient and paramedic by video. The authors believe this is the first report of a physician providing medical guidance to paramedics resuscitating an out-of-hospital cardiac arrest using these technologies.

Telemedicine is the use of a variety of digital, telephonic, or radio communications to allow for the remote (when distance separates) exchange of medical information.^1^ Telemedicine use in health care has seen dramatic growth after the coronavirus-19 pandemic, and its use in emergency medical services (EMS) appears to be increasing.^2^^,^^3^ However, there remain challenges with existing technology and communication infrastructure to provide a robust, Health Insurance Portability and Accountability Act compliant system allowing for real-time or near real-time data sharing, remote vital-sign monitoring, and reliable video transmission.^4^^,^^5^ These challenges are magnified in rural locales^4^ and were particularly highlighted during the recent emergency (911) cellular communication infrastructure collapse.^6^

Mayo Clinic Ambulance Service (MCAS) is a large, multistate EMS agency encompassing 15 ground and 3 rotor-wing (including 1 dual rotor-fixed wing) sites across the upper Midwest (Minnesota and Wisconsin), Starting with a single ground health system-affiliated MCAS site in Rochester, Minnesota, the EMS agency partnered with a veteran owned telemedicine company (OPTAC-X), which provides telemedicine solutions connecting physicians to medics in the field by video. Mayo Clinic Ambulance Service has 22,000 average annual 9-1-1 calls for service. The telemedicine system is unique in that it leverages a head-mounted, high-resolution (4K) camera with a visual heads-up display connected through wireless fidelity (WiFi) to a satellite communication (SATCOM) terminal mounted externally on top of an ambulance, allowing for hands-free paramedic interaction with a remotely located medical control physician. The goal of these telemedicine systems is to digitally connect with physicians to the homes or scenes of patients at time-of-injury or near time-of-illness in order to help reduce the cognitive burden of paramedics caring for complex patients, improve clinical outcomes, and provide awareness to connected physicians regarding what has occurred before patient transport and hospital arrival.

To the authors’ knowledge, this represents the first reported case of a medically-complex patient experiencing an out-of-hospital cardiac arrest where field paramedics used this novel combination of technologies (head-mounted camera using satellite communications in a patient’s home) to digitally bring an emergency medicine (EM)/EMS physician to the team and patient with subsequent coordination of a preliminary report to the emergency department (ED) code team preparing to receive the patient.

## Case

A 65-year-old woman patient suffered an out-of-hospital cardiac arrest at a residence approximately 4 miles from the receiving ED. Initial dispatch report to the responding EMS crew indicated their response was for a female having difficulty breathing with crew updated en route that cardiopulmonary resuscitation (CPR) was being initiated by a family member. Both fire department (basic life support) and EMS (advanced life support) assets were dispatched to the residence. Estimated downtime before CPR initiation was brief as the family reported hearing the patient fall in the bathroom. The family began chest compressions under the direction of the emergency medical dispatchers and continued CPR until the first responders arrived. The responding EMS crew consisted of 3 paramedics. On EMS arrival, the patient was both pulseless and apneic. Key timelines for the patient resuscitation are listed in Table.

**Table:** Resuscitation Timeline

15:19	911 Call—cardiopulmonary resuscitation (CPR) ongoing by family members
15:21	Ambulance and fire assets en route to patient
15:26	Arrival to patient
15:27	Telemedicine initiated
15:29	Assessment shows pulseless electrical activity; CPR started by fire Department using Lund University Cardiopulmonary Assist System device
15:30	Interosseous (IO) access to left tibia; bag mask ventilation initiated and I-Gel airway (size 5) placed
15:30-15:39	Continued CPR. No shock advised. Blood glucose obtained (127 mg/dL). One milligram epinephrine by IO
15:39	Endotracheal intubation by Glidescope and 6.5 mm endotracheal tube
15:39	Medical control physician recommends nebulized albuterol, sodium bicarbonate, and magnesium sulfate
15:41	Return of spontaneous circulation; acute rise in EtCO2 (54); vitals: BP 184/68, HR 124
15:44	50 mEq sodium bicarbonate administered by IO
15:47	Transport
15:48	Four milligrams magnesium sulfate initiated by infusion
15:53	Two milligrams midazolam administered for sedation
15:56	Emergency department arrival

BP, blood pressure; HR, heart rate.

Family on-scene reported the patient had a history of asthma and were concerned she may have had an asthma attack after descending a flight of stairs immediately before she collapsed. As such, paramedics were suspicious of a respiratory cause of the cardiopulmonary arrest. During the resuscitation, the patient remained in pulseless electrical activity on the monitor, and as such, no defibrillation was ever delivered. An EM/EMS physician provided assistance to the crew by the OPTAC-X telemedicine platform, provided recommendations beyond the already delivered advanced cardiac life support protocol, such as nebulized albuterol sulfate through the endotracheal tube, intravenous magnesium, and bicarbonate. The physician observed the field endotracheal intubation and visualized crews easily ventilating the patient with bag mask ventilation after endotracheal tube placement without resistance as would be anticipated with a severe asthma exacerbation. The patient regained spontaneous circulation in the field shortly after intubation and was transported to the ED. The physician made contact with the ED resuscitation team and provided full details on patient name and date of birth to allow for preliminary review of patient medical record, as well as timeline and details of prehospital resuscitation. The patient was ultimately admitted to the intensive care unit, extubated 48 hours later, and transferred to a general medical floor. A broad differential for the cause of her pulseless electrical activity arrest was considered but the exact etiology was never discovered. Because of the nature of this narrative case report, any patient identifying information has been withheld and patient consent was not required to be obtained.

### Technology and Data

As a pilot project to improve and enhance field telemedicine for paramedics treating complex and unstable patients in the prehospital setting, MCAS partnered with OPTAC-X, a company providing a complete end-to-end solution with its software-as-a-platform (SaaP). The platform integrates a wearable assisted reality video device (Realwear Inc) with a SATCOM terminal (Kymeta Corporation) and its software partner Vantiq (Walnut Creek). These technologies are managed through OPTAC-X’s artificial intelligence based network operations center and its artificial intelligence enhanced SaaP for field telemedicine in remote or austere locations.

As this is a pilot, no images or videos were taken or stored. OPTAC-X’s global connectivity solution utilizes the Kymeta terminal, which uniquely transmits data signals through both cellular and satellite platforms, automatically switching between them when data transmission slows. During this 10-minute telemedicine encounter, 54.25 megabytes of audio and video were uploaded through cellular and 4.92 megabytes by satellite.

This system has been tested and used by the United States Special Operations Command and the United States Army Special Operations Command special operations forces teams overseas, with successful reports of connectivity in austere locations in Africa. The ongoing pilot at Mayo Clinic is assessing the feasibility and reliability of this system within a civilian EMS system that serves both urban and rural locations.

**Figure 1:**
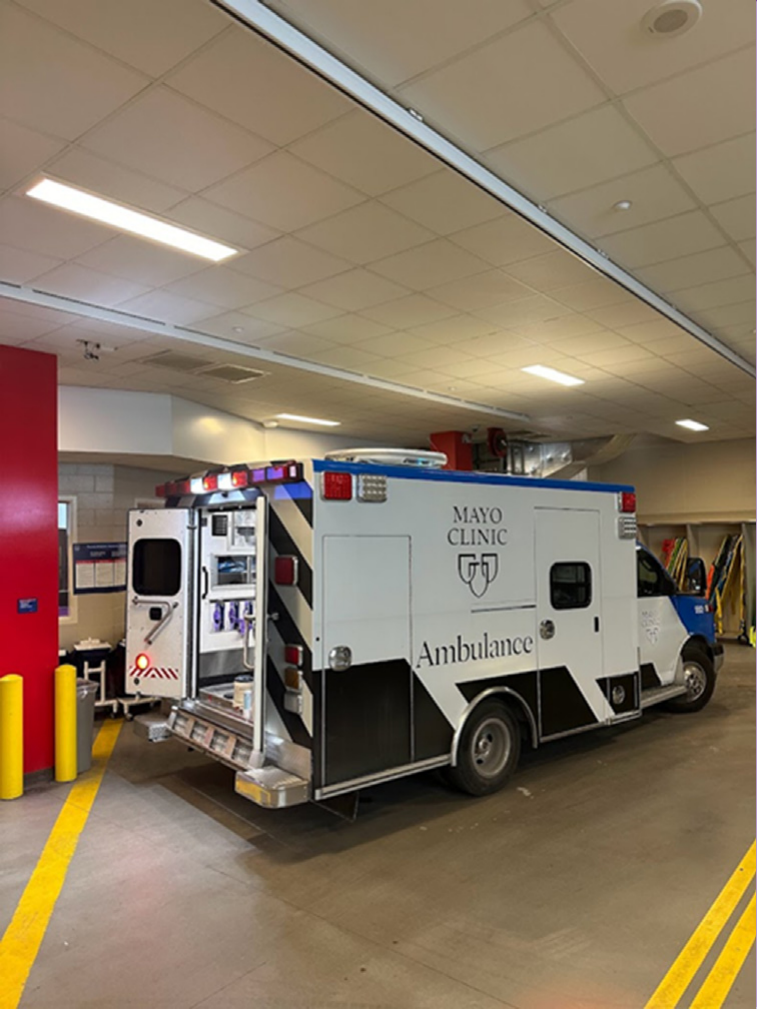
Satellite communication terminal on ambulance.

**Figure 2:**
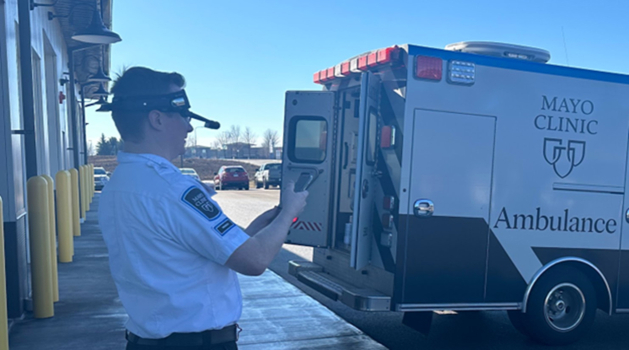
Paramedic wearing headset.

Paramedics control the headsets (Figure 1) using voice commands to connect with on-call medical control dual EM/EMS physicians through the OPTAC-X SaaP, which uses Zoom’s secure video telecommunication platform. Zoom was chosen for its adherence and certifications for privacy practices. Through the OPTAC-X integrated platform with Zoom, physicians can share videos or documents to the assisted reality devices for paramedic review, enabling them to review procedures or protocols before arriving at the scene. The successful handling of a cardiac arrest case in the basement of a patient’s home illustrates the novel combination of technologies maintaining communication even when the paramedic is away from the vehicle-mounted SATCOM terminal (Figure 2).

During this case, the Kymeta SATCOM terminal connected to the SES-3 satellite, a geosynchronous satellite positioned at the 103° west orbital slot, located approximately 35,782.9 km from the Earth’s surface. The terminal’s revolutionary electronically steered antenna allows for data transmission with near-zero latency both while in motion and stationery (on-scene.) This adaptive best path selection continually scanned satellites and local cellular platforms for the optimal connectivity to maintain audio and video for end-users. The method for selecting the best path was customized for this case, ensuring the most efficient and reliable connectivity. The latency management algorithm prioritized the fastest connection based on the latency of the second or third network hops. It periodically evaluated all healthy connections, directing new traffic to the link with the lowest latency to optimize performance for this latency-sensitive application during the cardiac arrest.

### Discussion

Demand for prehospital care services in the United States is significant with over 30 million patients requesting 911 service for medical care in 2020.^7^ A substantial percentage of those calls are rural with long transport times.^8^^,^^9^

Medical control physicians having robust visual and audio communication on-scene can help ease the cognitive load of paramedics by offering alternate thoughts in complex cases, overseeing low volume, high risk procedures, and prepare receiving clinical teams and resources with better information before arrival. This is especially important in rural areas with limited or nonexistent cellular connectivity with long. In addition, the ability to see patients along with paramedic teams should lower the risk and liability on paramedic teams in situations where patients refuse treatment or do not require ambulance transport. We also see significant potential for this technology to be used in community paramedic programs bringing physicians online and aiding in decision-making for return to the hospital, wound care, or patient education clarification.

To date, most prehospital cases utilizing video telemedicine center around telestroke and involve a fixed camera located internally within the ambulance.^10^, ^11^, ^12^, ^13^, ^14^ Little work has been done exploring the use of telemedicine dismounted from the ambulance for medical resuscitations or motor vehicle collisions. Within our own EMS system, we have previously attempted to deploy a telemedicine system using a portable camera and microphone (Teledoc Health, Purchase) connected by a cellular hotspot. However, this technology routinely failed because of either the inability of the cellular hotspot to connect to regional towers or limited bandwidth to support video connection. The unique aspect of this case is the novel combination of technologies allowing the remotely located physician the ability to provide real-time guidance to the paramedic crew by video with near-zero latency in connectivity, while simultaneously allowing the paramedics to remain hands-free as they continuously provide patient care.

Historically, when paramedics need to contact medical control for care recommendations or orders, this has one of the crew members stepping away from patient care to use a radio or phone for communication, thus removing them from direct patient care and requiring them to describe what they see to the physician. This loss of a single team member from a traditional 2-person EMS crew during a high-acuity situation can have detrimental impacts on patient care.^15^

Unique to this case, the paramedic utilizing the headset with medical control was separated from the ambulance and satellite communications terminal (in the patient’s basement) and was able to remain connected by local WiFi.

Most mobile cellular services are currently building their respective fifth-generation (5G) networks; and 5G-capable systems claim to have peak upload speeds of nearly 10 gigabits per second, whereas fourth-generation systems peak at 150 megabits per second (Mbps).^16^ To upload 4K video, communications systems need approximately 50 Mbps for unbroken video.^16^ However, upload speeds are often less than peak, and in a head-to-head test published in January 2023, the mobile carrier, T-Mobile, reportedly had the fastest upload speed at 17.9 Mbps.^17^

Having redundancy or alternate communication options for prehospital care teams is critical as health care advances toward a future with increased telemedicine capabilities through video and remote monitoring. Recently, the federal government’s FirstNet cellular band that runs on AT&T’s system for first responders went down,^18^ along with 911 systems in 4 states,^6^ effectively shutting down prehospital communication and putting millions of patient lives at risk. In rural or more austere locations, cellular service may be weak or absent, prohibiting any cellular data transmission.

In summary, we present the first case of prehospital management of out-of-hospital cardiac arrest using this novel combination of technologies. The use of a head-mounted camera allowing for the paramedic to remain hands-free while caring for the patient, WiFi connectivity to a satellite communications terminal, and video connectivity with an EM physician opens the door for future research in out-of-hospital resuscitative needs and complex care being delivered by prehospital clinicians in urban and rural locales.

## Potential Competing Interest

Dr Russi is in a “Know-How” agreement with OPTAC-X via Mayo Clinic Ventures. Dr Skeens reports receiving payments from Mayo Clinic for attending and traveling to meetings and serves as the Emergency Medicine Residents Association representative to the American College of Emergency Physicians Health Innovations and Technology Committee, an unpaid position. Dr Fullerton is the Chief Executive Officers of OPTAC-X. The other authors report no competing interests.
