# Supplementary material for: Use of a Head-Mounted Assisted Reality, High-Resolution Telemedicine Camera and Satellite Communication Terminal in an Out-of-Hospital Cardiac Arrest
Source: Mayo Clin Proc Digit Health. 2024 Oct 9;2(4):584–9. doi: 10.1016/j.mcpdig.2024.09.002 (PMC11975819; doi:10.1016/j.mcpdig.2024.09.002)
Supplement: Supplementary Data [file mmc1.pdf]

Mayo Proceedings Digital Health,

I give my permission for the use of my photos for the OPTAC Cardiac Arrest paper written by Dr. Russi. Please let me know if you have any questions.

Thanks,

9/27/2024

Jon Romans

701-426-8563
